# Severe Patients With ARDS With COVID-19 Treated With Extracorporeal Membrane Oxygenation in China: A Retrospective Study

**DOI:** 10.3389/fmed.2021.699227

**Published:** 2021-10-20

**Authors:** Wei Lai, Shuanglei Li, Zhongtao Du, Xinhua Ma, Junyu Lu, Wei Dong Gao, Geoffrey W. Abbott, Zhaoyang Hu, Yan Kang

**Affiliations:** ^1^Department of Critical Care Medicine, West China Hospital, Sichuan University, Chengdu, China; ^2^Division of Adult Cardiac Surgery, Department of Cardiology, The Sixth Medical Center of Chinese PLA General Hospital, Beijing, China; ^3^Center for Cardiac Intensive Care, Beijing Anzhen Hospital, Capital Medical University, Beijing, China; ^4^Department of Intensive Care Unit, Xiangya Hospital, Central South University, Changsha, China; ^5^Intensive Care Unit, The Second Affiliated Hospital of Guangxi Medical University, Nanning, China; ^6^Department of Anesthesiology and Critical Care Medicine, Johns Hopkins University School of Medicine, Baltimore, MD, United States; ^7^Bioelectricity Laboratory, Department of Physiology and Biophysics, School of Medicine, University of California, Irvine, Irvine, CA, United States; ^8^Laboratory of Anesthesiology & Critical Care Medicine, Translational Neuroscience Center, West China Hospital, Sichuan University, Chengdu, China

**Keywords:** coronavirus (COVID-19), severe acute respiratory syndrome, extracorporeal membrane oxygenation (ECMO), China, management

## Abstract

**Background:** The novel coronavirus disease 2019 (COVID-19) pandemic has become a global health crisis affecting over 200 countries worldwide. Extracorporeal membrane oxygenation (ECMO) has been increasingly used in the management of COVID-19-associated end-stage respiratory failure. However, the exact effect of ECMO in the management of these patients, especially with regards to complications and mortality, is unclear.

**Methods:** This is the largest retrospective study of ECMO treated COVID-19 patients in China. A total of 50 ECMO-treated COVID-19 patients were recruited. We describe the main characteristics, the clinical features, ventilator parameters, ECMO-related variables and management details, and complications and outcomes of COVID-19 patients with severe acute respiratory distress syndrome (ARDS) that required ECMO support.

**Results:** For those patients with ECMO support, 21 patients survived and 29 died (mortality rate: 58.0%). Among those who survived, PaO_2_ (66.3 mmHg [59.5–74.0 mmHg] and PaO_2_/FiO_2_ (68.0 mmHg [61.0–76.0 mmHg]) were higher in the survivors than those of non-survivors (PaO_2_: 56.8 mmHg (49.0–65.0 mmHg), PaO_2_/FiO_2_ (58.2 mmHg (49.0–68.0 mmHg), all *P* < 0.01) prior to ECMO. Patients who achieved negative fluid balance in the early resuscitation phase (within 3 days) had a higher survival rate than those who did not (*P* = 0.0003).

**Conclusions:** In this study of 50 cases of ECMO-treated COVID-19 patients, a low PO_2_/FIO_2_ ratio before ECMO commencement may indicate a poor prognosis. Negative fluid balance in the early resuscitation phase during ECMO treatment was a predictor of increased survival post-ECMO treatment.

## Background

Coronavirus disease 2019 (COVID-19) is a newly emerging disease caused by the novel SARS-CoV-2 virus. It was first reported in Wuhan, China in December 2019 and soon it spread all over the world (1). The World Health Organization (WHO) declared the COVID-19 a pandemic in March 2020. It is now affecting 213 countries and territories globally. As of April 2021, more than 193 million cases have been identified with over 2.9 million fatalities. Typical coronavirus infection causes respiratory symptoms. Common signs and symptoms include fever, cough, shortness of breath, fatigue, and dyspnea (2). Many COVID-19 patients have mild-to-moderate symptoms. However, elderly patients and those with existing chronic medical conditions, for example, diabetes, hypertension, chronic liver, and kidney disease, etc., are at higher risk of serious illness that may require intensive care unit (ICU) admission and are predisposed to severe acute respiratory distress syndrome (ARDS) (3). It has been shown that ARDS contributes to a mortality rate of 50% in ICU patients (4). A very recent report further demonstrated that 15% of patients infected with SARS-CoV-2 develop ARDS, with a resultant mortality rate in this group of 61.5% (5, 6).

The general treatment recommendations for ARDS from WHO interim guidelines indicate that ECMO may serve as a potentially life-saving strategy, providing circulatory and pulmonary support for patients with ARDS (7). During the 2009 influenza A (H1N1) winter pandemic, ECMO was used in treating H1N1–associated respiratory failure (8); however, because of insufficient evidence of decreasing mortality rate, the beneficial effect of ECMO remains controversial (9). Although a few reports have indicated the use of ECMO in the current pandemic (10–12), the exact role of ECMO in the management of COVID-19 is unclear. Importantly, ECMO is a very expensive and highly resource-demanding form of life-rescuing support. Therefore, recognizing and accurately selecting ECMO-appropriate candidates with SARS-CoV-2 pneumonia-associated ARDS would optimize the utilization of limited medical resources.

The principal aim of the study was to describe the clinical features, ECMO-related variables, technical characteristics of extracorporeal management, complications, and outcome of patients with COVID-19-associated end-stage respiratory failure who were treated with ECMO. To the best of our knowledge, this is the largest retrospective study of ECMO-treated COVID-19-induced ARDS in China.

## Methods

### Study Design and Patients

This retrospective study was approved by the institutional ethics committee of the West China Hospital, Sichuan University (ID: 2020-717). The institutional ethics review boards at all other participating centers approved the study protocol. All committees waived the need for informed consent because this is a non-interventional retrospective study and only electronic health records were used to extract data.

In this retrospective observational study, we included all adult COVID-19 patients (age from 35 to 91) from Beijing, Sichuan, Guangxi, Hunan, and Hebei province in China who received ECMO support between February 3, 2020, and January 23, 2021. Diagnosis of COVID-19 was confirmed by the use of real-time reverse transcription-polymerase chain reaction (RT-PCR) kits. All these critically ill patients were confirmed SARS-CoV-2 virus-positive and the severity of illness for COVID-19 patients was defined according to the Chinese Clinical Guidance for COVID-19 Pneumonia Diagnosis and Treatment (version 7.0) (13). ARDS was diagnosed according to the Berlin definition (14). If the PaO_2_/FiO_2_ ratio of the ARDS patient was <150 mmHg, the lung protective strategies and the prone position were applied. Patients who were unresponsive to conventional ARDS rescuing therapies were eligible for ECMO support if they met the following clinical criteria: (1) developed a refractory severe ARDS; (2) Lung Injury Murray Score ≥ 3;(3) developed uncompensated hypercapnia with pH <7.25 or PaCO_2_ > 60 mmHg over 6 h; (4) PaO_2_/FiO_2_ < 80 over 6 h; (5) PaO_2_/FiO_2_ <50 mm Hg over 3 h. Patients with refractory severe multiorgan failure and/or significant neurological injury were excluded.

After initiating ECMO, all the patients received an optimal lung-protective strategy, i.e., FiO_2_ was set to 40–70% to maintain SaO_2_ over 90 or PaO_2_ over 60 mmHg, tidal volume 4–6 ml/kg ideal body weight, Pplat 25–30 cmH_2_O, PEEP 5–15 mmH_2_O, respiratory rate 4–10 breaths per minute, with transpulmonary driving pressure <14 cmH_2_O.

We used heparin as an anticoagulant therapy during ECMO treatment. Heparin was continuously infused intravenously at 5–25 ug/kg.h. Therapeutic strategies were defined as the activated clotting time **(**ACT**)** of 180–200 s, the activated partial thromboplastin time (**a**PTT) of 40–60 s. ACT and aPTT were monitored every 2–4 h daily.

### Data Collection

A case report form was used for data collection by experienced clinicians. Two researchers cross-checked the collected data to ensure data accuracy and integrity. We obtained the following retrospective data from electronic medical records: demographic data, including age, gender, body mass index (BMI); predefined comorbidities, including high blood pressure, diabetes, and hyperthyroidism; pre-ECMO laboratory data, including hemoglobin, leukocyte, CREA, alanine aminotransferase (ALT), and total bilirubin. Sequential Organ Failure Assessment (SOFA) score was calculated in both groups within 24 h after admission. We also recorded information on the timing of onset of symptoms, commencement of mechanical ventilation, and ECMO treatment. Data on baseline lung functions, including pH, PO_2_, PaO2/FiO_2_, PCO_2_, and lactate before ECMO placement were also recorded. Ventilator settings, e.g., mode, FiO_2_, positive end-expiratory pressure (PEEP), respiratory rate, tide volume, and plateau pressure (Pplat) were recorded either prior to or after ECMO initiation. We collected ECMO-related treatment details, including; (1) ECMO mode, ECMO duration, and ventilation mode during ECMO support; (2) whether patients received protective mechanical ventilation, were placed in a prone position, had bronchoalveolar lavage and daily sputum suction, and/or used vasoactive drugs. We also collected data of PaO_2_ and PaCO_2_ after 24 h of ECMO treatment. Complications associated with ECMO, the characteristics of mortality cases, and the causes of death were documented.

The primary outcomes were survival rate or mortality. Patients who died during ECMO treatment were classified in the latter category, as were those who were weaned off ECMO but died within 48 h post-ECMO (three patients). The 21 patients who were successfully discharged after ECMO treatment were classified as survivors.

### Statistical Analysis

Continuous variables were expressed as median with interquartile range (IQR) and were compared using Wilcoxon rank-sum tests. Categorical variables were reported as frequency and percentage. Fisher's exact test was employed to compare the number of patients falling into one of the two ECMO survivor or non-survivor groups. Statistical analyses and survival curves were generated using GraphPad Prism 5.0 (GraphPad Software, Inc., San Diego, California, USA). *P* < 0.05 was used to indicate statistical significance.

## Results

### General Characteristics of Patients Treated With ECMO

**Figure 1:**
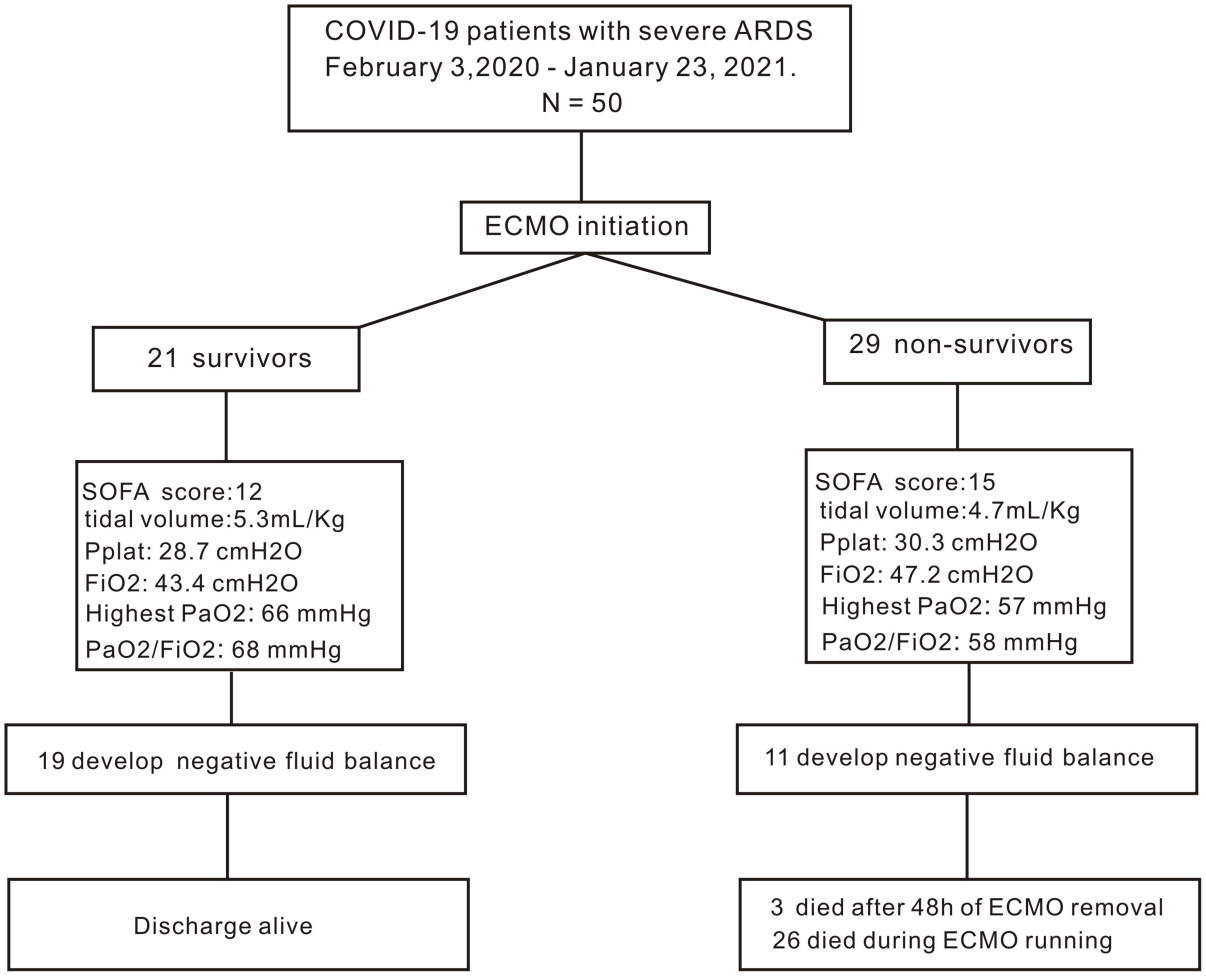
Flow diagram of the study. PaO_2_, arterial partial pressure of oxygen; PaO_2_/FiO_2_, the ratio of arterial partial pressure of oxygen to fraction of inspired oxygen; FiO_2_: the fraction of inspired oxygen.

The study population comprised 50 confirmed patients with COVID-19 who were admitted to the hospital between February 3, 2020, and January 23, 2021, and were placed on ECMO (Figure 1). Table 1 depicts patient demographic data of both survivors and non-survivors, as well as their laboratory examinations after hospitalization. The median age of these patients was 65.3 years (IQR: 58.8–73.0), ranging from 35 to 91 years. Although not statistically significant, the patients in the non-survivor group had a higher age as compared with the survivor group. A total of 34 male patients and 16 female patients were included in the study, accounting for 68 and 32% of the total patient population, respectively. The median body mass index was 25.3 (IQR: 23.1–27.8) and was not different between survivors and non-survivors. Various comorbidities were investigated in the current study. The most common associated predefined comorbidities were high blood pressure and diabetes mellitus in 22 patients (44%), and 15 patients (30%), respectively. There were no statistically significant differences between survivors and non-survivors regarding the incidence of high blood pressure (*P* = 0.0857) or diabetes mellitus (*P* = 0.21). As for laboratory tests, all the patients developed leukocytosis, with an increased WBC count of 13.5 × 10^9^/L (IQR: 11.5–15.8 × 10^9^/L). SARS-CoV-2 coronavirus also attacks red blood cells, resulting in a reduced hemoglobin concentration (104.1 g/l, IQR: 88.0–126.0 g/l). The creatinine level was within the normal range. Liver damage was seen in all the patients infected with COVID-19, as evidenced by elevated levels of ALT and total bilirubin. Meanwhile, we did not find any difference between the survivors and non-survivors with respect to blood chemistry (all *P* > 0.05).

**Table 1:** General characteristics of patients with extracorporeal membrane oxygenation.

**Parameters**	**Total** **(*n =* 50)**	**Survivors** **(*n =* 21)**	**Non-survivors** **(*n =* 29)**	***P*-value**
Age	65.3 (58.8–73.0)	61.2 (54.5–70.0)	68.2 (62.0–75.0)	0.07
Gender:male, *n* (%)	34 (68.0)	12 (57.1)	22 (75.9)	0.22
Gender:female, *n* (%)	16 (32.0)	9 (42.9)	7 (24.1)	0.22
Body Mass Index (BMI)	25.3 (23.1-27.8)	25.8 (23.3–29.0)	25.0 (22.7–27.4)	0.48
High blood pressure, *n* (%)	22 (44.0)	6 (28.6)	16 (55.2)	0.09
Diabetes, *n* (%)	15 (30.0)	4 (19.0)	11 (37.9)	0.21
Leukocyte (× 10^9^/L)	13.5 (11.5–15.8)	13.3 (11.8–15.7)	13.7 (11.2–16.3)	1
Hemoglobin (g/L)	104.1 (88.0–126.0)	108.5 (97.0–129.0)	100.9 (86.0–125.0)	0.16
CREA (μmol/L)	60.9 (36.8–71.9)	68.6 (39.0–74.5)	55.3 (35.7–70.5)	0.29
Alanine aminotransferase (U/L)	42.7 (22.0–54.3)	45.8 (27.5–61.5)	40.4 (22.0–53.0)	0.27
Total bilirubin (μmol/L)	24.1 (11.9–32.8)	20.5 (12.5–27.5)	26.7 (11.3–35.0)	0.64

*p-values: survivors vs. non-survivors*.

### Characteristics of Patients and Mechanical Ventilation Protocol Before Initiation of ECMO

Details of the severity of illness before initiation of ECMO are shown in Table 2. Generally, all these COVID-19 patients developed severe ARDS with deteriorated lung function. The SOFA score was calculated within 24 h after admission. The mean SOFA score was higher in non-survivors than that in survivors (*P* < 0.0001). PaO_2_ was low in all the patients with a median of 60.8 mmHg (IQR: 52.0–70.0 mmHg) and was lower in the non-survivors (56.8 mmHg, IQR: 49.0–65.0 mmHg) when compared with survivors (66.3 mmHg, IQR: 59.5–74.0 mmHg, *P* = 0.0033). Accordingly, the median PaO_2_/FiO_2_ ratio was 62.3 mmHg (IQR: 52.0–73.3 mmHg), and survivors (68.0 mmHg, IQR: 61.0–76.0 mmHg) had significant higher PaO_2_/FiO_2_ ratio as compared with the non-survivors (58.2 mmHg, IQR: 49.0–68.0 mmHg, *P* = 0.0051). All the patients had retention of carbon dioxide in the blood, with no statistically significant differences between non-survivors and survivors (*P* = 0.34). Both the survivors and non-survivors had low pH. Serum lactate was slightly elevated with a median of 2.3 mmol/l in survivors and a median of 3.2 mmol/l in non-survivors (*P* = 0.35). The mechanical ventilator settings before ECMO treatment are listed in Table 2. Generally, there were no differences in the ventilator settings (i.e., PEEP, Pplat, and ventilation mode) between survivor and non-survivor groups (all *P* > 0.05). Although not achieving *P* < 0.05, there was a trend toward a shorter time between the onset of symptoms and initiation of mechanical ventilation in the survivors vs. non-survivors (11.8 days, IQR: 5.0–16.5 vs.14.9 days, IQR: 4.5–21.0 days, respectively; *P* = 0.54) or the initiation of ECMO (15.5 days, IQR: 7.5–21.0 vs. 20.4 days, IQR: 6.5–27.0 days, respectively; *P* = 0.38), as well as the duration between the start of mechanical ventilation and ECMO (3.8 days, IQR: 1.0–5.0 vs. 6.2 days, IQR: 1.5–11.0 days, respectively; *P* = 0.38).

**Table 2:** Lung function and treatment before the commencement of extracorporeal membrane oxygenation.

**Parameters**	**Total** **(*n =* 50)**	**Survivors** **(*n =* 21)**	**Non-survivors** **(*n =* 29)**	***P*-value**
SOFA score	13.7 (12.0–15.0)	12 (11–14)	1 (514–16)	<0.0001
Highest PaO_2_, mmHg	60.8 (52.0–70.0)	66.3 (59.5–74.0)	56.8 (49.0–65.0)	0.0033
PaO_2_/FiO_2_, mmHg	62.3 (52.0–73.3)	68.0 (61.0–76.0)	58.2 (49.0–68.0)	0.0051
Highest PaCO_2_, mmHg	60.2 (54.0–67.6)	57.6 (47.0–67.8)	62.1 (55.1–68.6)	0.34
Lowest PH	7.3 (7.2–7.3)	7.3 (7.2–7.4)	7.3 (7.2–7.3)	0.97
Lactate, mmol/L	2.9 (1.8–3.1)	2.3 (1.8–2.9)	3.2 (1.8–3.8)	0.35
Highest PEEP, cm H_2_O	11.4 (10.0–12.0)	11.7 (10.0–12.5)	11.2 (10.0–12.0)	0.53
Pplat, cm H_2_O	30.5 (26.0–34.0)	29.3 (24.5–33.5)	31.3 (28.0–35.0)	0.5
Choice of ventilation mode of P/C	23 (46.0)	9 (42.9)	14 (48.3)	0.78
Choice of ventilation mode of A/C	27 (54.0)	12 (57.1)	15 (51.7)	0.78
Duration of onset of symptoms to MV, median (IQR), d	13.6 (5.0–20.3)	11.8 (5.0–16.5)	14.9 (4.5–21.0)	0.54
Duration of onset of symptoms to ECMO, median (IQR), d	18.3 (7.0–25.3)	15.5 (7.5–21.0)	20.4 (6.5–27.0)	0.38
Duration of MV to ECMO, median (IQR), d	5.2 (1.0–9.3)	3.8 (1.0–5.0)	6.2 (1.5–11.0)	0.38

*MV, mechanical ventilation; Pplat, airway plateau pressure; PEEP, positive end expiratory pressure; P/C, pressure control mode; A/C, volume control mode; p-values: survivors vs. non-survivors*.

### Extracorporeal Membrane Oxygenation Treatment Details

As shown in Table 3, the median duration of ECMO support was 17.9 days (IQR: 6.0–22.0 days), and no difference was found between survivors and non-survivors (*P* = 0.95). All the patients received lung-protective strategy after ECMO initiation in our study. Veno-venous (VV) ECMO was used in 94.0% of patients with the rest (6.0%) of the patients treated with veno-arterial (VA) ECMO. Regarding mechanical ventilator settings, pressure control (P/C) ventilation mode was used in 62.0% of patients during ECMO treatment, while 38.0% of patients have ventilated in volume control (A/C) mode. There were no differences in ventilation modes between survivors and non-survivors (P/C: *P* = 0.26; A/C: *P* = 0.26, respectively). Meanwhile, there is no difference between survivors and non-survivors regarding the respiration rate (*P* = 0.25), driving pressure (*P* = 0.15), and the highest PEEP (*P* = 0.16). However, the FiO_2_ was lower (*P* = 0.04), and tidal volume was higher in survivors (*P* = 0.014), accompanied by the reduced Pplat value (*P* < 0.0001) indicating that the patients with better lung compliance had better survival rate. After 24 h of ECMO treatment, significant improvement in oxygenation was observed in all patients as evidenced by increased PaO_2_ (93.1 mmHg, IQR: 75.8–109.3 mmHg). Survivors appeared to have higher PaO_2_ than non-survivors, although this difference did not reach statistical significance (*P* = 0.3). Accordingly, the average PaCO_2_ decreased to 38.5 mmHg (IQR: 34.5–42.5 mmHg) after ECMO treatment. However, no difference in PaCO_2_ was found between survivors and non-survivors (*P* = 0.79). Thirty-nine patients (78%) were subjected to prone positioning during ECMO treatment (90.5% survivors and 69.0% non-survivors, *P* = 0.09). We also studied the influence of fluid balance and found that within the first 3 days of ECMO, more survivors achieved negative fluid balance (19 survivors, 90.5% vs. 11 non-survivors, 37.9%, *P* = 0.0003). During the entire ECMO treatment period, patients underwent a series of therapies, including daily sputum suction (23 out of 50 patients, 46%) and bronchoalveolar lavage (39 out of 50 patients, 78.0%). A combination of vasoactive drugs during ECMO offered no discernible benefit to the patients (*P* = 1).

**Table 3:** Lung function and treatment details after commencement of ECMO.

**Parameters**	**Total** **(*n =* 50)**	**Survivors** **(*n =* 21)**	**Non–survivors** **(*n =* 29)**	***P*-value**
ECMO running days	17.9 (6.0–22.0)	19.1 (7.5–24.0)	17.0 (5.5–22.0)	0.95
Initial ECMO mode of VV, *n* (%)	47 (94.0)	21 (100)	26 (89.7)	0.25
Initial ECMO mode of VA, *n* (%)	3 (6.0)	0 (0.0)	3 (10.3)	0.25
choice of ventilation mode of P/C during ECMO treatment, *n* (%)	31 (62.0)	11 (52.4)	20 (69.0)	0.26
choice of ventilation mode of A/C during ECMO treatment, *n* (%)	19 (38.0)	10 (47.6)	9 (31.0)	0.26
Respiration rate, BPM	10.3 (10.0–10.0)	10.5 (10.0–11.0)	10.1 (10.0–10.0)	0.25
driving pressure, cm H_2_O	13.5 (13.0–14.0)	13.7 (13.0–14.0)	13.4 (13.0–14.0)	0.15
Highest PEEP, cm H_2_O	8.8 (8.0–10.0)	8.6 (8.0–9.0)	9.0 (8.0–10.0)	0.16
tidal volume, ml/kg	4.9 (4.4–5.6)	5.3 (4.8–6.0)	4.7 (4.0–5.0)	0.014
Pplat, cm H_2_O	29.6 (29.0–30.0)	28.7 (28.0–30.0)	30.3 (30.0–31.0)	<0.0001
FiO_2_, mmHg	45.6 (40.0–50.0)	43.4 (40.0–50.0)	47.2 (40.0–50.0)	0.04
Highest PaO_2_ after 24 h of ECMO treatment, mmHg	93.1 (75.8–109.3)	96.9 (79.0–116.3)	90.3 (74.3–105.0)	0.3
Highest PaCO_2_ after 24 h of ECMO treatment, mmHg	38.5 (34.5–42.5)	38.6 (34.0–44.0)	38.3 (34.3–42.0)	0.79
Prone positioning during ECMO treatment, *n* (%)	39 (78.0)	19 (90.5)	20 (69.0)	0.09
Development of negative fluid balance at the end of the first 72 h of ECMO treatment, *n* (%)	30 (60.0)	19 (90.5)	11 (37.9)	0.0003
Daily sputum excretion during ECMO treatment, *n* (%)	23 (46.0)	9 (42.9)	14 (48.3)	0.78
Bronchoalveolar lavage during ECMO treatment, *n* (%)	39 (78.0)	17 (81.0)	22 (75.9)	0.74
Using vasoactive drugs during ECMO treatment, *n* (%)	43 (86.0)	18 (85.7)	25 (86.2)	1

*P/C, pressure control mode; A/C, volume control mode; VV, veno–venous mode; VA, veno–arterial mode; Pplat, airway plateau pressure; PEEP, positive end expiratory pressure; p-values: survivors vs. non-survivors*.

### Complications and Patient Outcomes

As shown in Table 4, in our study, of the 50 patients studied, 21 (42.0%) were weaned from ECMO successfully and were discharged. The total mortality rate was 58.0% (29 out of 50 patients). Twenty-six of the non-surviving patients died when they were on ECMO support (89.7%), while three died during the first 48 h of ECMO decannulation (10.3%). The median duration between onset of symptoms and death was 38.2 days (IQR: 25.5–45.0 days). Primary reasons for death were bleeding (9 out of 29 deaths, 31.0%), respiratory failure (4 out of 29 deaths, 13.8%), sepsis (14 out of 29 deaths, 48.3%), multiple organ failure (18 out of 29 deaths, 62.1%), and heart failure (4 out of 29 deaths, 13.8%). ECMO-related complications are presented in Table 4. Briefly, of the 50 patients, 13 had decreased platelet counts (26.0%), 39 exhibited bleeding (78.0%), 16 had infections (32.0%), three exhibited thrombus (6.0%), and three exhibited pneumothorax (6.0%).

**Table 4:** Complications of patients treated with extracorporeal membrane oxygenation.

**Characteristics of mortality**	
Mortality, *n* (%)	29 (58.0)
death during ECMO running, *n* (% of total death)	26 (89.7)
death after 48 h of ECMO removal, *n* (% of total death)	3 (10.3)
Duration of onset of symptoms to die, median (IQR), d	38.2 (25.5–45.0)
**Cause of death**, ***n*** **(% of the total 29 death)**	
Bleeding, *n* (%)	9 (31.0)
Respiratory failure, *n* (%)	4 (13.8)
Sepsis, *n* (%)	14 (48.3)
Multiple organ failure (MOF), *n* (%)	18 (62.1)
Heart failure, *n* (%)	4 (13.8)
**Complications**, ***n*** **(% of the total 50 patients)**	
Decreased platelet counts	13 (26.0)
Bleeding	39 (78.0)
Infection	16 (32.0)
Thrombus	3 (6.0)
Pneumothorax	3 (6.0)

## Discussion

The current COVID-19 pandemic has now infected more than 137 million people worldwide. Approximately 15–30% of COVID-19 patients developed severe respiratory compromise. Mortality for patients with COVID-19-related ARDS is substantial (15). ECMO is an external artificial lung device and a life-saving rescue strategy for patients with severe lung disease (7). However, the use of ECMO remains controversial for the following reasons: it requires highly specialized medical staff, bears high economic costs and limited ECMO resources, and is associated with a high risk of potentially lethal complications, including bleeding, infection, or thrombus (16).

Although ECMO remains controversial in patients with severe ARDS, ECMO has been used previously to treat virus-induced respiratory failure, such as H1N1 flu in 2009 (8). While the efficacy of ECMO in the setting of COVID-19 has been unclear, the resemblance of COVID-19 to seasonal virus influenza-related respiratory syndrome suggests possible benefits for the use of ECMO in COVID-19 patients with severe and refractory respiratory failure, as it aims to provide sufficient oxygen to the body and helps the lungs to recover (17). At the time of writing, the Extracorporeal Life Support Organization (ELSO) showed that 8080 suspected or confirmed COVID-19 patients received ECMO support, with 52% discharged alive globally (by July 2021).

In our study, the patient population comprised 50 confirmed COVID-19 patients who were admitted to the hospital and were placed on ECMO. The pathological features of patients suffering from the COVID-19 viral disease resemble those observed in Middle Eastern Respiratory Syndrome (MERS) and Severe Acute Respiratory Syndrome (SARS) coronavirus infection (18), all of which were characterized by severe pulmonary fibrosis and inflammation. Patients with severe COVID-19 meet the ARDS Berlin definition criteria with respect to the symptoms of pulmonary depression and severity of hypoxemia (14). The criteria for the initiation of ECMO include the levels of PaO_2_ and PaO_2_/FiO_2_ ratio, i.e., PaO_2_/FiO_2_ <80 mmHg for over 6 h or PaO_2_/FiO_2_ <50 mmHg for over 3 h.

We found in our study that patients who had lower SOFA scores and better oxygenation, as reflected by higher PaO_2_ and PaO_2_/FiO_2_ ratios (> 70 mmHg) before ECMO treatment, were more likely to survive. Meanwhile, during the lung-protective mechanical ventilation, survivors who had better pulmonary compliance were associated with a larger tide volume and lower plateau pressure. In addition, there was a trend toward a shorter duration of onset of symptoms to mechanical ventilation and mechanical ventilation to ECMO in the survivors, albeit these differences did not achieve statistical significance because of the finite patient availability. A possible explanation for this difference is that ECMO-treated non-survivors typically had the more severe disease when compared with ECMO-treated survivors, thus, perhaps needed greater rescue efforts and longer time. Therefore, although our data may provide evidence that earlier exertion of respiratory intervention techniques may result in better outcomes, these findings need to be substantiated with larger patient cohorts.

Fluid balance is a strategy for balancing fluid output and input of the patients. Clinicians and researchers have already realized the negative impact of fluid overload in patients admitted to ICUs. It has been shown that altered fluid balance is associated with increased mortality and worse clinical outcomes in patients with acute kidney injury (19), septic shock (20), and lung injury (21). During ECMO, large-volume intravenous fluid infusions are required, especially in the early resuscitation phase to maintain sufficient ECMO blood flow (22). Studies have also shown that proper fluid management may improve patient outcomes (23). However, there is no established optimal fluid balancing protocol for ECMO patients, and debates exist regarding the impact of positive (fluid output is lower than the input) vs. negative (fluid volume deficit) fluid balance on mortality in critically ill patients. A majority of studies showed the beneficial effect of negative fluid balance. For example, in a large cohort study including 1,000 patients with lung injury, a negative fluid balance was shown to be associated with improved lung function (24). Moreover, Schmidt et al. also demonstrated that the patients with negative fluid balance had a higher survival rate than those with positive fluid balance in adult patients treated with ECMO (23). Negative fluid balance was, however, previously found to be associated with increased mortality in ICU patients (25). In addition, a positive fluid balance was found to be associated with increased hospital mortality in adult patients treated with ECMO (23, 26). To the best of our knowledge, no previous studies have addressed the issue of fluid management regarding the critically ill COVID-19 patients treated with ECMO. Our study is the first to describe the effects of fluid balance in the COVID-19 patient treated with ECMO and we found that there was a significant difference in achieving negative fluid balance in the early resuscitation phase (within 3 days of resuscitation) between survivor and non-survivors (*P* = 0.0003). Future large-cohort prospective or retrospective studies on the impact of negative fluid balance in the long-term ECMO treatment (over 3 days) will provide further invaluable information on this subject.

There are several limitations to our study. First, our study of 50 ECMO-treated patients cannot prevent type 2 errors due to the small sample size and limited statistical power. Second, we only collected fluid information during the early part of the resuscitation phase; therefore, whether negative fluid balance affects mortality during the long-term ECMO treatment remains unknown. Moreover, we are not certain whether VV and VA ECMO subgroups may have responded differently to fluid therapy because that portion of this study is underpowered to detect meaningful differences between these two subgroups.

## Conclusion

This multicenter, retrospective study demonstrates that pre-ECMO low PO_2_/FiO_2_ ratio indicates poor prognosis, as occurred in most cases of ECMO-treated non-survivors with the COVID-19. Meanwhile, more survivors achieved negative fluid balance in the early resuscitation phase during the ECMO treatment than non-survivors did.

## Data Availability Statement

The original contributions presented in the study are included in the article/supplementary material, further inquiries can be directed to the corresponding author/s.

## Ethics Statement

The studies involving human participants were reviewed and approved by West China Hospital, Sichuan University. The Ethics Committee waived the requirement of written informed consent for participation.

## Author Contributions

WL, SL, ZH, and YK conceptualized the paper. WL, SL, ZD, XM, JL, and YK collected the data. WL and ZH conducted data analysis. ZH wrote the initial draft. WL, SL, ZD, XM, JL, and YK helped to revise the manuscript. WG and GA revised manuscript critically for important intellectual content. All authors read and approved the final manuscript.

## Funding

This project was supported by the Project of Novel Coronavirus Pneumonia in West China Hospital (HX2019nCoV027 to YK).

## Conflict of Interest

The authors declare that the research was conducted in the absence of any commercial or financial relationships that could be construed as a potential conflict of interest.

## Publisher's Note

All claims expressed in this article are solely those of the authors and do not necessarily represent those of their affiliated organizations, or those of the publisher, the editors and the reviewers. Any product that may be evaluated in this article, or claim that may be made by its manufacturer, is not guaranteed or endorsed by the publisher.
